# Mining metagenomes reveals diverse antibiotic biosynthetic genes in uncultured microbial communities

**DOI:** 10.1007/s42770-023-00953-z

**Published:** 2023-03-28

**Authors:** Dina H. Amin, Wedad M. Nageeb, Amr Elkelish, Rabab R. Makharita

**Affiliations:** 1 Microbiology Department, Faculty of Science, Ain Shams University, Cairo, 11566, Egypt; 2 Medical Microbiology and Immunology Department, Faculty of Medicine, Suez Canal University, Ismailia, 41111, Egypt; 3 Biology Department, College of Science, Imam Mohammad ibn Saud Islamic University (IMSIU), 90950, Riyadh, 11623 Saudi Arabia; 4 Department of Biology, College of Sciences and Arts, Khulis, University of Jeddah, Jeddah, 21959, Saudi Arabia; 5 Botany and Microbiology Department, Faculty of Science, Suez Canal University, Ismailia, 41522, Egypt

**Keywords:** Bioinformatic tools, Metagenomic library, NRPS, Phylogenetic analysis, Antimicrobials

## Abstract

Pathogens resistant to antimicrobials form a significant threat to public health worldwide. Tackling multidrug-resistant pathogens via screening metagenomic libraries has become a common approach for the discovery of new antibiotics from uncultured microorganisms. This study focuses on capturing nonribosomal peptide synthase (NRPS) gene clusters implicated in the synthesis of many natural compounds of industrial relevance. A NRPS PCR assay was used to screen 2976 *Escherichia coli* clones in a soil metagenomic library to target NRPS genes. DNA extracts from 4 clones were sequenced and subjected to bioinformatic analysis to identify NRPS domains, their phylogeny, and substrate specificity.

Successfully, 17 NRPS-positive hits with a biosynthetic potential were identified. DNA sequencing and BLAST analysis confirmed that NRPS protein sequences shared similarities with members of the genus *Delftia* in the *Proteobacteria* taxonomic position. Multiple alignment and phylogenetic analysis demonstrated that clones no. 15cd35 and 15cd37 shared low bootstrap values (54%) and were distantly far from close phylogenetic neighbors. Additionally, NRPS domain substrate specificity has no hits with the known ones; hence, they are more likely to use different substrates to produce new diverse antimicrobials. Further analysis confirmed that the NRPS hits resemble several transposon elements from other bacterial taxa, confirming its diversity. We confirmed that the analyses of the soil metagenomic library revealed a diverse set of NRPS related to the genus *Delftia.* An in-depth understanding of those positive NRPS hits is a crucial step for genetic manipulation of NRPS, shedding light on alternative novel antimicrobial compounds that can be used in drug discovery and hence supports the pharmaceutical sector.

## Background

According to a report released by the World Health Organization (WHO), the number of new antibiotics in development is insufficient to relieve the growing threat of antimicrobial resistance [1]. WHO warns against the urgent need for new antibiotics to tackle antimicrobial resistance. WHO advised more investments in basic science, drug discovery, and clinical development. As most agents currently in the pipeline are modifications of existing antibiotic classes, urgent solutions are needed to beat multidrug-resistant pathogens [2–4].

Usually, research focuses on bacterial isolates that can grow in vitro and thus lack the 99% of bacteria that remain uncultivated [5, 6] or even harbor cryptic gene clusters that are not documented [6, 7]. Metagenomics has developed as an alternative approach to conventional microbial screening that allows comprehensive screening of microbial genomes in their natural environments. Metagenomics is a molecular approach used to analyze the microbial community residing in a particular environment, which overcomes the limitations of culture-based methods [8]. The metagenome represents a capture of the microbial population through the extraction of its DNA at a given time point [9]. This approach allows for the rapid and precise discovery of novel genes, proteins, and even complete genomes of noncultivable organisms compared to traditional microbiology and molecular techniques [9]. The utilization of metagenomic libraries in screening strategies has recently gained prominence in the study of biosynthetic gene cluster diversity and as a tool for drug discovery [10].

The screening of large metagenomic libraries has been shown to detect numerous potentially interesting clones [11, 12]. Metagenomics is supported by the integration of computational methods including BLAST (Basic Local Alignment Search Tool) algorithms to obtain a list of related hits with a certain annotation which can also be used to exploit taxonomic information and metabolic potential of microorganisms. Bioactive peptides, such as antimicrobial peptides, produced by microorganisms have opened a new area of research and expanded the use of antimicrobial peptides as an alternative to traditional antibiotics to combat drug resistance. The study of metagenomics has improved the search for bioactive peptides by increasing our understanding of the metabolic abilities of microorganisms, leading to the discovery and identification of new microorganisms with potential to produce these peptides. Additionally, metagenomics provides information about the taxonomy of microorganisms and their evolutionary relationships and helps to understand the distribution of bioactive peptides in different environments. As a result, metagenomics has increased the pool of potential sources for bioactive peptides with various biological activities [9, 13].

The synthesis of bioactive peptides produced by microorganisms through the ribosomal and nonribosomal mechanisms is well known. The synthetic route of nonribosomal peptides is an alternative means of producing highly specialized polypeptides [14]. Numerous nonribosomal peptides create secondary metabolites such as antibiotics, antifungals, toxins, anticancer drugs, siderophores, and immunosuppressants [15, 16]. Nonribosomal peptide synthetases (NRPSs) are modular mega-synthases that catalyze nonribosomal peptide assembly from protein and nonprotein amino acids [17, 18]. NRPSs consist of an adenylation (A) domain, condensation (C) domain, and a peptidyl carrier protein (PCP) as a minimum core structure [19, 20]. Criteria such as composition, number, and arrangement of elongation modules in the NRPS system command the mass and chemical structure of the produced natural compounds.

Several studies confirmed that mutations in NRPS genes and phylogenetically far domains in the modular organization of NRPS lead to a functional complex that can generate new bioactive molecules [21–23]. Additionally, the DNA sequence of the NRPS gene cluster could be used to predict the chemical nature of peptides; consequently, it correlates to the chemical nature and the biological function of the compounds produced via the NRPS biological system [18, 24]. Therefore, it is crucial to study the phylogenic insight of NRPS in the potential actinomycete that would provide new opportunities for drug discovery [21, 25, 26].

The present study aims to screen the soil fosmid metagenomic library to target biosynthetic pathways. Based on the evidence, fosmid libraries evaluate the diversity of biosynthetic gene clusters and help in finding several new bioactive compounds [27, 28]. NRPS PCR assay and DNA sequencing were used to capture NRPS biosynthetic gene clusters from the environmental metagenomic DNA library recovered from different sites in Cuba [28, 29]. Further, molecular and bioinformatic analysis of the positive NRPS hits retrieved from the metagenomic library is considered a preliminary step for the manipulation of these genes in heterologous hosts [11, 12].

## Materials and methods

### The metagenomic DNA source and routine cultivation

Thirty-one plates (96-well) of metagenomic fosmid library constructed from Cuban soil were kindly provided by Prof. Elizabeth Wellington, The University of Warwick. The Cuban library included 2976 clones, with an average insert size of approximately 35 kb, for a total library size of 104 Mb. All plates were recovered in replicas using fresh Luria-Bertani (LB) broth media containing chloramphenicol (35 mg/ml). An aliquot of 150 μl LB broth media containing chloramphenicol was placed in each well of the plates. An amount of 15 μl metagenomic DNA was transferred from each well individually of old plates into new plates with fresh LB broth media using an electronic micropipette. Each plate was covered with a permeable sheet and incubated at 37°C for 24 hours. All plates were held at 4°C.

### Extraction of environmental DNA from E. coli clones in metagenomic libraries

Using a multichannel pipette, a volume of 150 μl LB broth containing 12.5 mg/ml of chloramphenicol was added to each well of 96-well culture plates (Becton Dickinson Labware). Every well was inoculated with 15 μl of *E. coli* clones. Plates were then tapped and incubated at 37 °C for 24 h. 10 μl from every 96-well was transferred into a 500 μl Eppendorf tube using a multichannel pipette. Centrifugation of each sample was carried out at 13,000 × *g* for 5 min. The supernatants were discarded, and the fosmid-bearing *E. coli* pellets were collected using the GeneJET plasmid miniprep package (QIAGEN). All steps are performed following the manufacturer’s instructions, and fosmid DNA was collected for PCR assay.

### NRPS PCR assay and screening of the fosmid metagenomic library

The environmental DNA metagenomic library, of about 104 Mb in size, was screened using polymerase chain reaction (PCR) for the identification of NRPS clusters with ADEdom5 and ADEdom3 primer pairs obtained from Sigma Company, Egypt, as shown in Table 1. PCR mixture included 12.5 μl PCR Master Mix (Promega, Madison, WI, USA), 6.25 μl of distilled water, 1.25 μl DMSO, 2 μl of 0.8 μM of each primer in 25 μl total volume, and 1 μl of 0.1 μM extracted fosmid DNA template. The PCR program was set as follows: 5 min at 95°C followed by 40 cycles of 1 min at 95°C, 1 min at 60°C, and 1.5 min at 72°C, followed by a final extension of 10 min at 72°C [30, 31]. Five microliters of every PCR product and 1 Kb ladder (Fermentus) were detected using agarose gel electrophoresis for 30 min at 90 V. *Streptomyces coelicolor* was positive control in all PCR reactions as it contains NRPS genes. The gel was stained with 50 mg/ml of ethidium bromide, and images of DNA bands of the predictable size were recorded using a UV transilluminator at a wavelength of 312 nm (Hercules, CA).

**Table 1:** Primer pair used for amplifying NRPS genes obtained from fosmid metagenomic library

Primers	Sequence (5^′^_3^′^)	Fragment (bp)	Reference
ADEdom5	5′-ACS GGC NNN CCS AAG GGC GT-3′	450	[30, 31]
ADEdom3	5′-CTC SGT SGG SCC GTA-3′	450	[30, 31]

### Purification and sequencing of PCR products

Bands of NRPS gene fragments recovered from four fosmid *E. coli* clones were purified from 1% agarose gel using the QIAquick Gel Extraction Kit (QIAGEN; Venlo, Netherlands). An amount of fifty microliters of each PCR sample was added to the 125 μl PB buffer according to the manufacturer’s instructions. Then, ten microliters of 3 M sodium acetate (pH 5.0) was included until the mixture became yellow. Samples were transferred to a QIAquick column and centrifuged at 17,900 × *g* for 60 sec, and then, the flow was removed. QIAquick columns were washed with 0.75 ml BE buffer and centrifugated for 60 sec at 17,900 × *g*. Each QIAquick column was set in a clean 1.5 ml microcentrifuge tube to elute the DNA, and then, 50 μl of BE buffer (10 mM Tris·Cl, pH 8.5) was carefully added to the center of the QIAquick membrane. The column was then centrifuged at 17,900 × *g* for 60 sec. Storage of the purified samples remained in a deep freezer at −20 ° C and was ready for sequencing. The sequencing of purified PCR products of NRPS gene fragments was conducted at GATC Biotech AG, Cologne, Germany [32]. All sequenced amplicons were placed under GenBank accession numbers.

### Comparative analyses of the obtained gene sequences via BLAST

Sequenced amplicons of selected strains were transformed into amino acid sequences to define open reading frames (ORF) using the ORF finder server (https://www.ncbi.nlm.nih.gov/orffinder/). Comparative analyses of the deduced amino acid sequences were conducted against corresponding nonribosomal peptide domain sequences in the GenBank database using the BLASTP algorithm (https://blast.ncbi.nlm.nih.gov/Blast). Multiple alignments of NRPS amino acid sequences against similar sequences were constructed using BioEdit software to represent the patterns of amino acids with similar descent or shared functional constraints [33–38]. Multiple alignments of NRPS nucleotide sequences of all clones were constructed in Geneious 9.1.8 software. Alignment bundles representing the percentage of nucleotide identity were represented as ribbons whose ends represent the extent of the set alignments drawn by Circos software (http://circos.ca/software/).

### Neighbor-joining phylogenetic tree construction

Neighbor-joining phylogenetic trees were built using translated nucleotides of NRPS clones versus the related amino acid sequences accessible in the NCBI database using BLASTP. Multiple sequence alignment of amino acid sequences was performed using the CLUSTAL W program [39] and neighbor-joining method within the Molecular Evolutionary Genetics Analysis (MEGA) software version 6.0 [30–38, 40–52]. The numbers at each branch’s node correspond to a percentage bootstrap value based on 1000 replicates. The scale bar showed the nucleotide sequence dissimilarity.

### Downstream analysis of the defined amino acid sequences

A comprehensive overview of NRPS sequence domains was predicted via the NRPS Predictive BLAST web server available at http://nrps.igs.umaryland.edu/blast.html. This web server uses hidden Markov model (HMM) to predict the identity of every domain in NRPS gene sequences based on a statistical representation of protein groups with similar sequences and hence the functional similarity. Additionally, substrate specificity of each NRPS clone was expected using the nonribosomal peptide synthase substrate predictor based on the NRPSsp database which is available at http://www.nrpssp.com/execute.php [53]. Additionally, the PDBsum pictorial database in Protein Data Bank (PDB) was also used to analyze the NRPS sequence against the sequences of all proteins in the PDB. The top hits are listed with links to their PDBsum pages showing molecules that make up the structure including protein chains, DNA, ligands, and metal ions (https://www.ebi.ac.uk/thornton-srv/databases/cgi-bin/pdbsum/GetPage.pl?pdbcode=index.html).

### Analysis of transposons in the recovered clones

A comprehensive overview of NRPS sequence domains was predicted via TnCentral (https://tncentral.ncc.unesp.br/), a specialized web resource for prokaryotic transposable elements (TE) [32]. It contains ∼400 carefully annotated TE, including transposons from several families. These TE include genes conferring transcription factors and genes involved in metabolism. In this study, we selected the transposon hits with the highest bit scores as they refer to the highest significance [32].

### Investigating the clone sequence similarity against NCBI metaproteome

All clone sequences were blasted against the metaproteome sequences in the NCBI database, which can give a more clear presentation of the functional annotation of these clones. Most similar hits were selected and were used to build a maximum likelihood phylogenetic tree with the default multiple sequence alignment methods, and the tree inference method was FastTree 2.1.1 builder in Geneious 9.1.8 software (http://www.geneious.com/). Branch lengths are proportional to nucleotide substitutions/site, and trees were visualized in iTOL v6.3 software (https://itol.embl.de/).

## Results

### PCR assay detection of NRPS gene cluster in fosmid metagenomic library

**Fig. 1:**
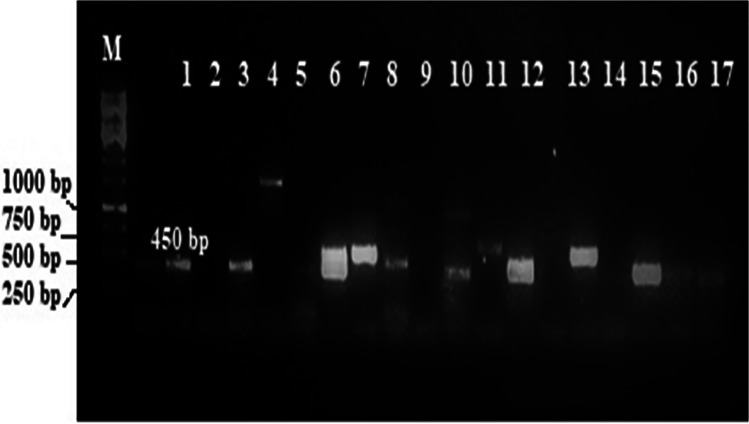
NRPS PCR screening of soil metagenomic fosmid library. PCR amplicons were subjected to electrophoresis in 1% agarose gels as previously described in the “Materials and methods” section. Lane M: 1 kb ladder, lane 1: control (*S. coelicolor*), lanes 3, 6, 8, 12, 15: positive hits of 450 bp of NRPS genes isolated from *E. coli* clones of the soil metagenomic library by ADEdom3/5 primers

NRPS PCR assay screening of 2976 clones of the soil metagenomic library retrieved a total of 17 positive clones thus putatively harboring NRPS biosynthetic pathway as in Fig. 1. Consequently, the hit rate can be calculated as the total DNA captured divided by the number of positive hits (17 hits); therefore, it is 1 in 6 Mb for the soil metagenomic library. This expects that the NRPS assay can detect an average greater than one gene cluster per 1.4 genomes; based on that, the average *E. coli* genome is 4.6 Mb in size. The sequenced clones primarily had similarities to sequences found in Gram-negative bacteria *Proteobacteria* including the genus *Delftia*. Selected sequenced clone amplicons can be viewed under GenBank accession numbers MT538186, MT538187, MT538188, and MT538189.

### Bioinformatic analysis DNA sequences obtained from E. coli fosmid clones

**Fig. 2:**
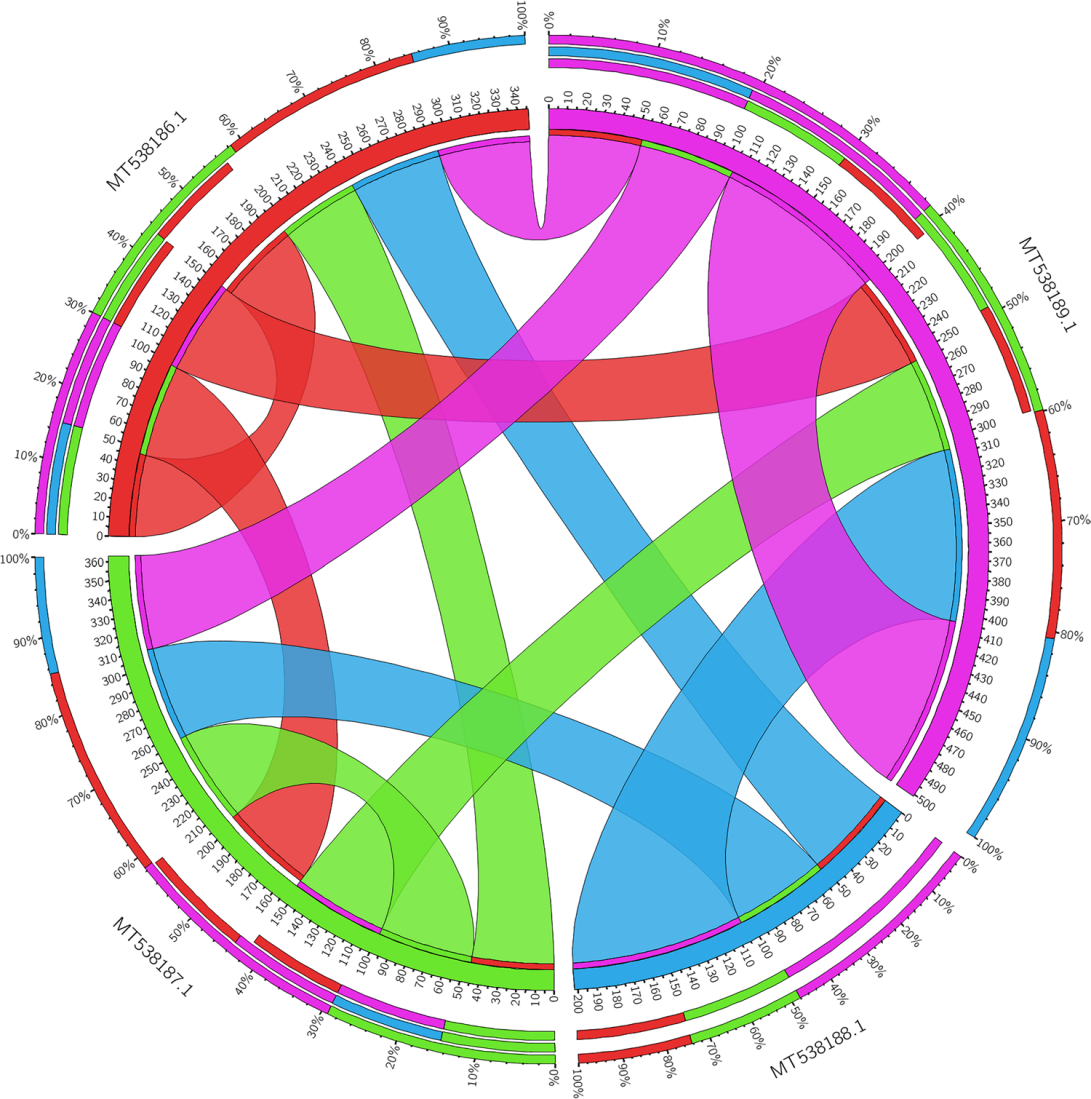
A chord diagram displaying the interrelationships between NRPS gene fragment of positive clone retrieved from soil metagenomic library. Alignment bundles were built up from clone sequences retrieved using default multiple sequence alignment methods in Geneious 9.1.8 software. Percentage of nucleotide identity was represented as ribbons whose ends represent the extent of the alignments drawn by Circos software

Bioinformatic investigations of ORF amino acid sequences of *E. coli* clones derived from metagenomic libraries revealed significant homology to the NRPS gene available in the NCBI database. Clones no. 15cd30 and 15cd34 showed (100%) blast identity with NRPS genes of *Delftia tsuruhatensis* and *Delftia acidovorans*, respectively. While NRPS gene sequences of clone 15cd35 and clone 15cd37 exhibited recognizable blast identity 98.82% and 95.24% with corresponding NRPS gene fragments of *Delftia acidovorans*, all sequences were not identical to each other as shown in Table 2. Multiple alignments of the nucleotide sequences obtained from the 4 clones showed that they share around 50% of the nucleotide identity. MT538188 and MT538189 showed high nucleotide identity with (97%), while MT538186 and MT538187 were different from each other and other sequences with 46–52%. Several repetitive DNA sequences were recorded in all clone sequences except MT538188 as shown in Fig. 2.

**Table 2:** Results of BLAST identity of the positive clones retrieved from soil metagenomic library using the BLASTP algorithm

Primer pairs	ORF	Size of product (aa)	Characteristics of homolog(s)				
			Protein ID	Deduced role	BLAST identity	Origin	Accession no. of the sequenced amplicon
15cd30	ORF5	127 aa	2347175	Nonribosomal peptide synthetase	100.00%	*Delftia tsuruhatensis*	MT538186
15cd34	ORF6	127 aa	2347190	AMP-binding protein in NRPS cluster	100%	*Delftia acidovorans*	MT538187
15cd35	ORF5	175 aa	2347562	AMP-binding protein	98.82%	*Delftia acidovorans*	MT538188
15cd37	ORF4	90 aa	2347577	AMP-binding protein	95.24%	*Delftia acidovorans*	MT538189

### Phylogenetic analysis of the detected NRPS gene fragments

**Fig. 3:**
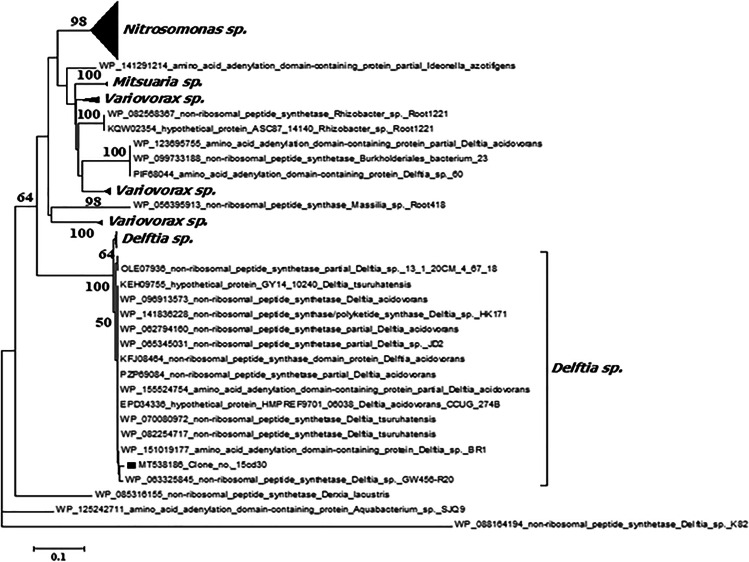
Phylogenetic tree based on the amino acid sequence of NRPS gene fragment of positive clone retrieved from soil metagenomic library. Multiple sequences were aligned using the CLUSTAL W program [39] against corresponding amino acid sequences. The tree was constructed using the neighbor-joining method using MEGA software version 6.0 [40]. The numbers beside the branches indicate the percentage bootstrap value of 1000 replicates. Bootstrap values greater than 50% are at the node. The scale bar indicates nucleotide sequence dissimilarity

**Fig. 4:**
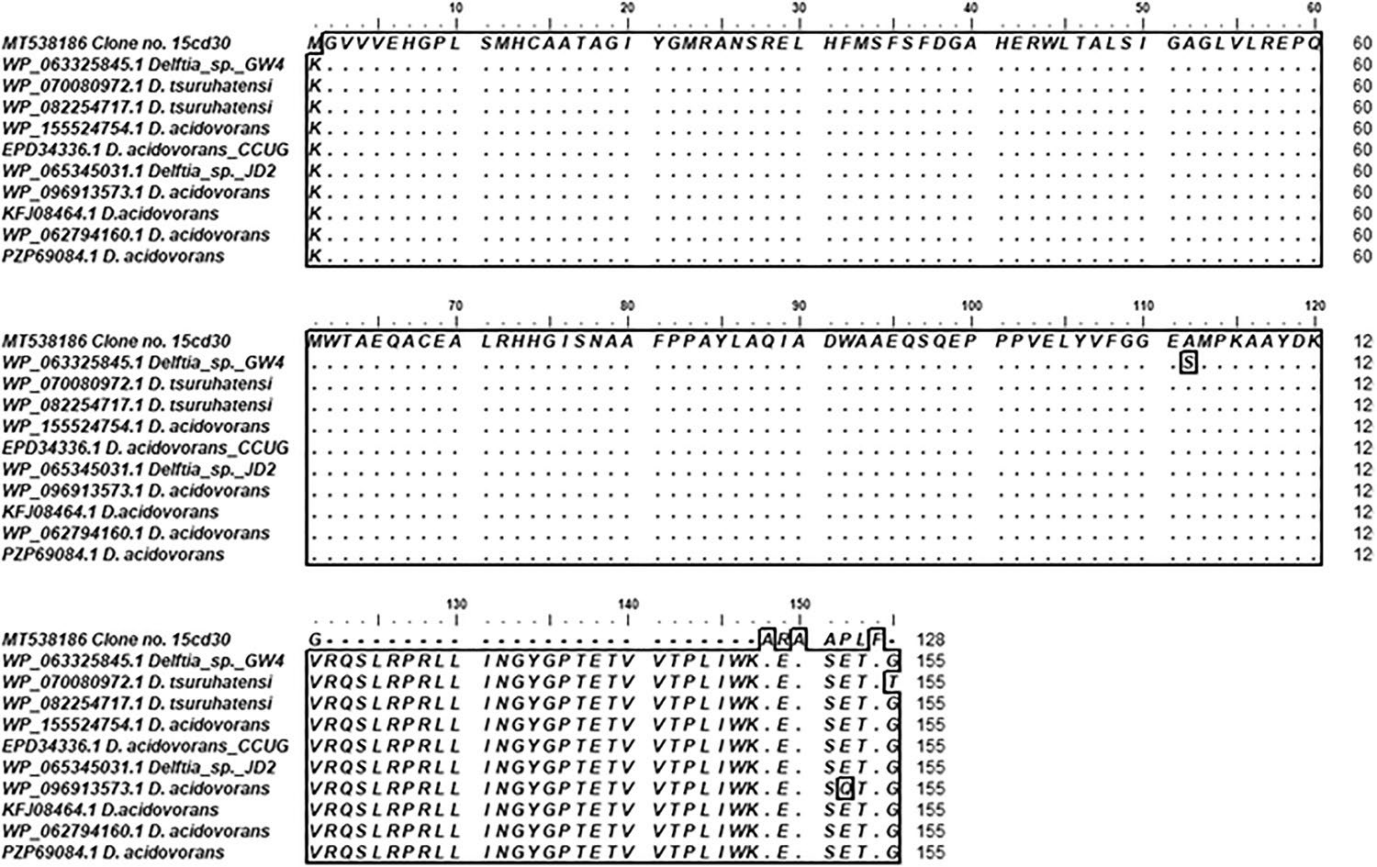
Amino acid sequence multiple alignment pattern for the identification of conserved motifs in NRPS-positive clone retrieved from soil metagenomic library. Multiple sequences were aligned using the CLUSTAL W program[39] against corresponding amino acid sequences using BioEdit program [33]. Conservation is viewed by plotting identities to the first sequence as dots with outlining

Phylogenetic analyses were carried out to locate metagenomic NRPS gene fragments inside the phylogenetic tree built with the NRPS published sequences on GenBank. Clone 15cd30 was clustered with *Delftia tsuruhatensis* (WP 082254717), *Delftia tsuruhatensis* (WP 070080972), *Delftia* sp. BR1 (WP 151019177), and *Delftia* sp. GW456-R20 (WP 063325845) with bootstrap value 100 as shown in Fig. 3. Additionally, amino acid sequence multiple alignment pattern of NRPS clone no. 15cd30 was identical to corresponding amino acid sequences of *Delftia tsuruhatensis* (WP 082254717), *Delftia tsuruhatensis* (WP 070080972), and *Delftia* sp. GW4 (WP063325845.1) at the core region of the NRPS domain, while at the terminal several mismatches were detected as presented in Fig. 4.

**Fig. 5:**
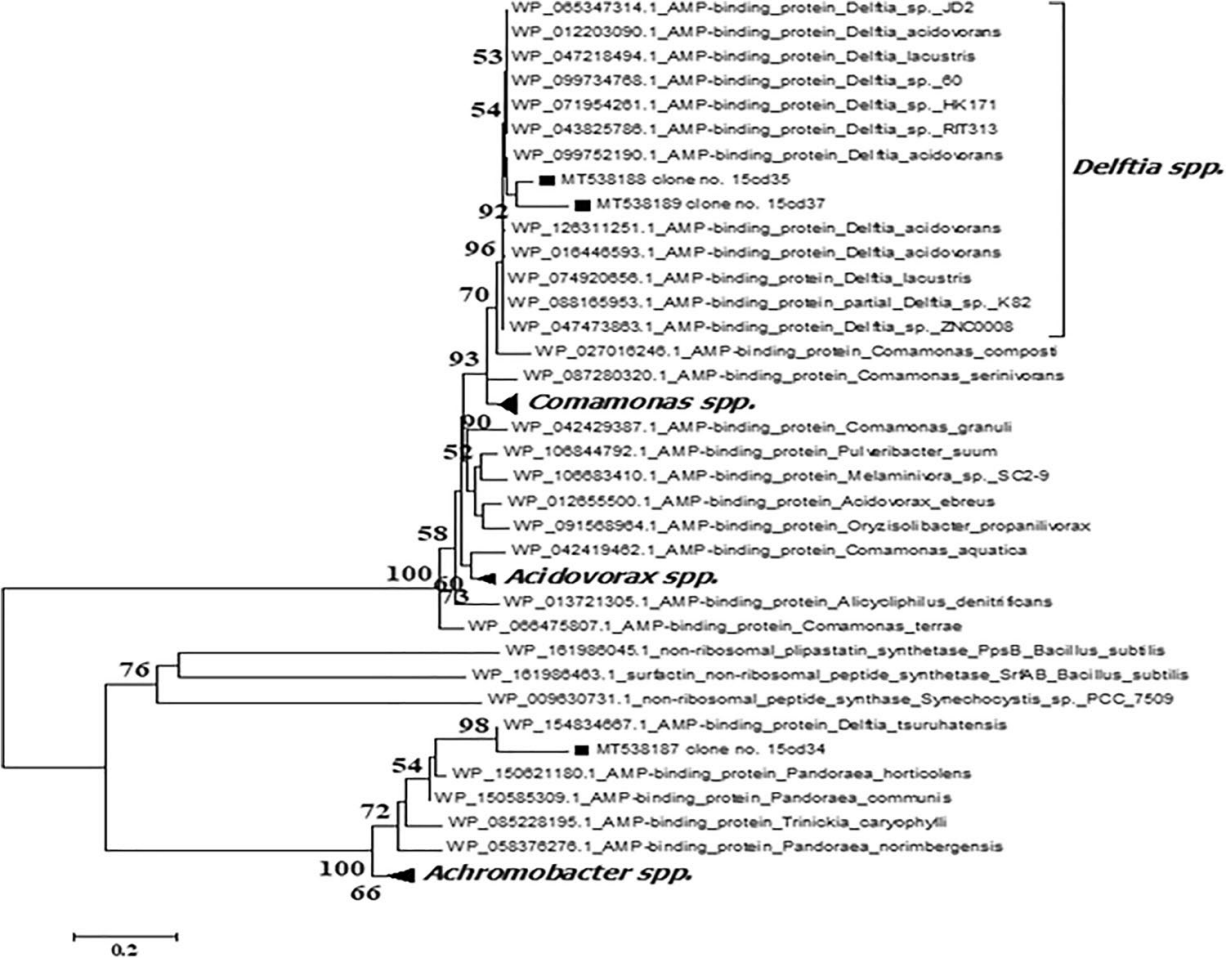
Phylogenetic tree based on the amino acid sequence of NRPS gene fragment of positive clones retrieved from soil metagenomic library. Multiple sequences were aligned using the CLUSTAL W program [39] against corresponding amino acid sequences. The tree was constructed using the neighbor-joining method using MEGA software version 6.0 [40]. The numbers beside the branches indicate the percentage bootstrap value of 1000 replicates. Bootstrap values greater than 50% are at the node. Scale bars indicate nucleotide substitutions per site

**Fig. 6:**
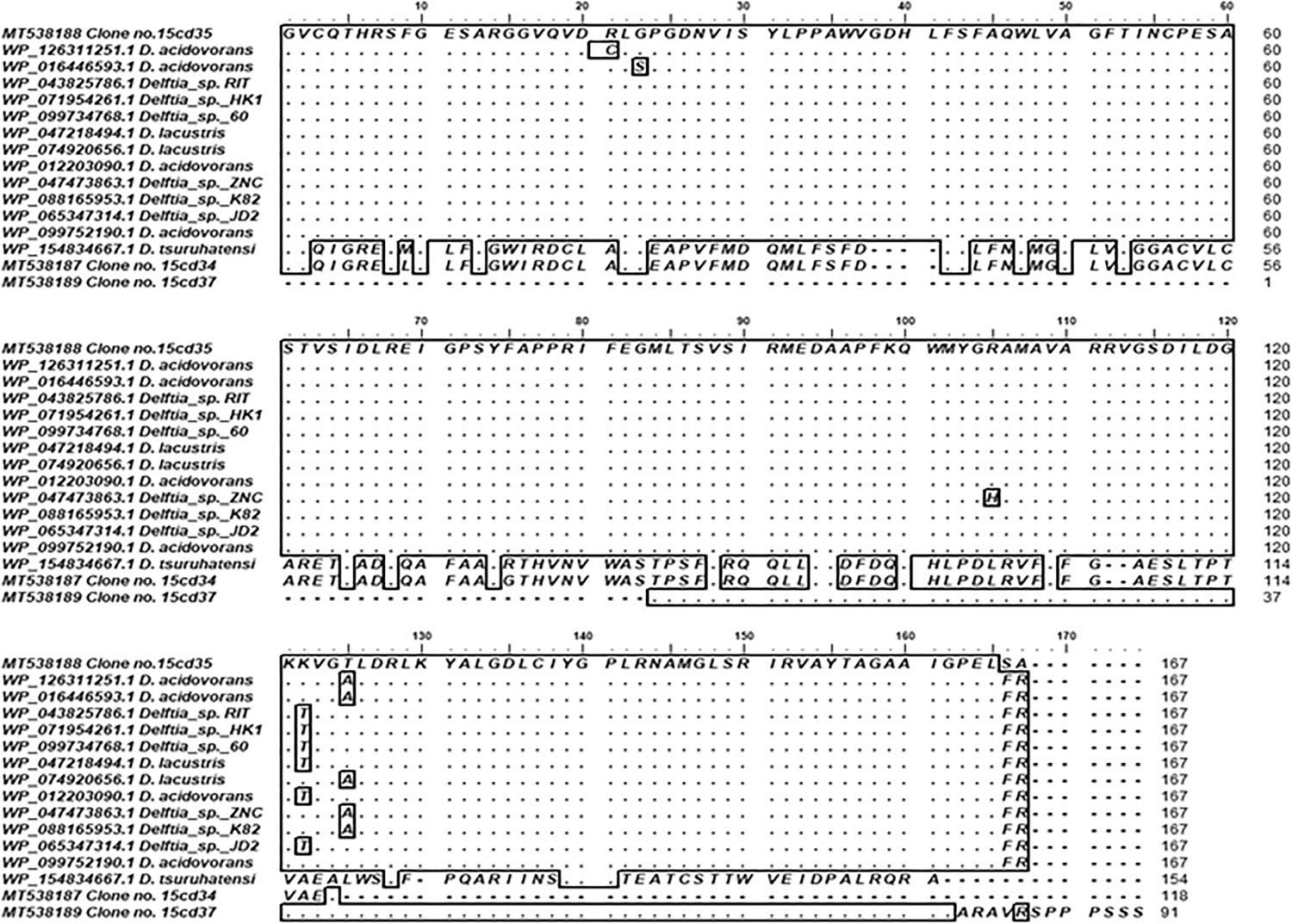
Amino acid sequence multiple alignment pattern for the identification of conserved motifs in NRPS-positive clone retrieved from soil metagenomic library. Multiple sequences were aligned using the CLUSTAL W program [39] against corresponding amino acid sequences using BioEdit program [33]. Conservation is viewed by plotting identities to the first sequence as dots with outlining

The closest match to the NRPS gene of clone no. 15cd34 is AMP-binding protein (within the NRPS gene cluster) was *Delftia tsuruhatensis* (WP 154834667.1). It is clustered with members of *Pandoraea* and *Achromobacter* genera with bootstrap value 100% as shown in Fig. 5. Amino acid sequence multiple alignment pattern of NRPS clone no. 15cd34 was identical to corresponding amino acid sequences of *Delftia tsuruhatensis* (WP 154834667.1) except for the terminal region as presented in Fig. 6.

Phylogenetic analysis confirmed that clone 15cd35 and clone 15cd37 were clustered in a separate clade from AMP-binding protein sequences (within NRPS) that belonged to members of the genus *Delftia* spp. within the b-*Proteobacteria*. *Delftia acidovorans* (WP 099752190.1) and *Delftia* sp. RIT313 (WP043825786.1) were identified as the nearest phylogenetic relative to both clones with a low bootstrap value (54%) as shown in Fig. 5. Amino acid sequence multiple alignment pattern of NRPS clone no. 15cd35 shows several amino acid substitutions to corresponding conserved regions of amino acid sequences of all corresponding *Delftia* spp*.* in the middle core region as presented in Fig. 6.

**Fig. 7:**
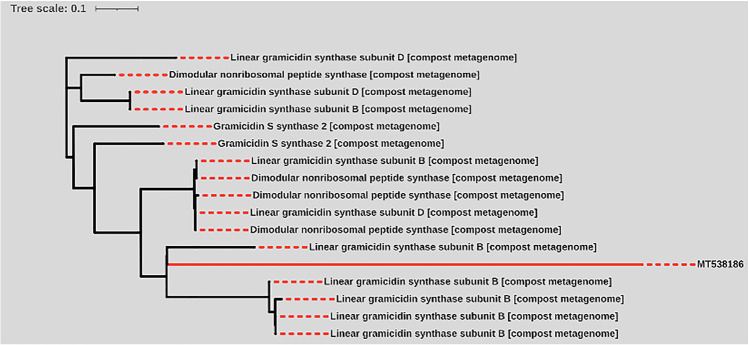
Phylogenetic tree based on the NRPS nucleotide sequence of positive clones retrieved from soil metagenomic library against metaproteome in the NCBI database. Maximum likelihood phylogenetic tree was built with the default multiple sequence alignment methods, and the tree inference method was FastTree 2.1.1 builder in Geneious 9.1.8 software. Branch lengths are proportional to nucleotide substitutions/site, and trees were visualized in iTOL v6.3 software

Prediction of NRPS clones was conducted using the PKS/NRPS analysis website. HMM hits of NRPS positive clones retrieved from the soil metagenomic library resolve diversity in NRPS domains as shown in Table 3. The substrate of NRPS clones retrieved from the soil metagenomic library was predicted by NRPSpredictor2 as presented in Table 4. The substrate for the NRPS adenylation domains of clone nos. 15cd30 and 15cd34 appears to be phenylalanine and alanine, respectively. No substrates were recorded to other NRPS domains of clones 15cd35 and 15cd37 as presented in Table 4. PDBsum bioinformatic tool demonstrated hits of all clones against all protein sequences and related 3D structures deposited in the Protein Data Bank as shown in Table 5. The bioinformatic analysis confirmed the presence of diverse NRPS domains in all sequences retrieved from the metagenomic library. Another point indicates that all clones showed similar hits with transposable elements in several taxa of bacteria, which is a sign of diversity that extends to different genera than *Delftia* genus (Table 6). Our results confirmed that only the clone 15cd30 sequence was grouped with a similar protein sequence retrieved from compost metagenome, with cluster coding for gramicidin (Fig. 7).

**Table 3:** List of top HMM hits of NRPS-positive clones retrieved from soil metagenomic library using NRPS analysis website

Clones	Protein identifier	Domain	Coordinates of the hit		Hit probability score
15cd30	ORF5	ER domain	1	127	7.2
15cd34	ORF6	DH domain	1	92	7.1
15cd35	ORF5	AT domain	1	131	7.4
15cd37	ORF4	KR domain	17	91	9.3

*Several domains of NRPS gene cluster with defined functions: *ER* enoylreductase, *DH* dehydratase, *AT* acyltransferase, *KR* ketoreductase

**Table 4:** Results of substrates that bind to given NRPS-positive clones retrieved from soil metagenomic library using nonribosomal peptide synthase substrate predictor based on HMM predictor

Clones	Protein identifier	Adenylation domain start position	Adenylation domain end position	Substrate name	Score
15cd30	ORF5	0	124	Phenylalanine	82.7
15cd34	ORF6	0	120	Alanine	128.2
15cd35	ORF5	–	–	ND	–
15cd37	ORF4	–	–	ND	–

*ND not detected

**Table 5:** Results of top hits of positive clones retrieved from soil metagenomic library against all protein sequences and related 3D structures deposited in the Protein Data Bank using the PDBsum database

Clones	ORF	PDB code	Amino acid overlap	*z*-score	Ligands	Protein name	Organism
15cd30	ORF5	5n9x	129	215.2	THR, ATP, 8QN	Adenylation domain thr1	*Streptomyces* sp. Oh-5093
15cd34	ORF6	3fce	118	382.8	ATP	d-Alanyl carrier protein ligase	*Bacillus cereus*
15cd35	ORF5	4oae	66	118.7	SO4, CLM, EDO	GNAT superfamily acetyltransferase	*Pseudomonas aeruginosa*
15cd37	ORF4	4oae	66	130.9	SO4, CLM, EDO	GNAT superfamily acetyltransferase	*Pseudomonas aeruginosa*

**Table 6:** Alignment of NRPS clones generated from metagenome library with transposons elements using TnCentral database

Clones sequence	TnCentral accession	TE name	Host organism	TE family	Position	Identity	Score (bits)	*E* value
15cd30	TnAmu1-AP012041	TnAmu1	*Acidiphilium multivorum* AIU301	Tn3	74–89	100%	32	0.26
15cd34	Tn3434-AY232820	Tn3434	*Paracoccus pantotrophus* DSM11027	Tn3	43–58	100%	32	0.30
15cd35	TnXax1.1-NC_016053	TnXax1.1	*Xanthomonas arboricola pv. pruni* CFBP 55306	Tn3	287–305	94%	30	2.4
15cd37	TnXax1.1-NC_016053	TnXax1.1	*Xanthomonas arboricola pv. pruni* CFBP 55306	Tn3	288–306	94%	30	1.2

## Discussion

Our environment still contains lots of undiscovered microorganisms that can support potential drug discovery [5]. In this study, the soil metagenomic library was screened to discover the biosynthetic pathways in uncultured microorganisms. Similarly, several studies have involved the mining of metagenomic libraries to determine the variety of biosynthetic gene clusters [10]. Screening of large biosynthetic gene libraries detected abundant remarkable clones with biosynthetic potential [11, 12, 28]. In this study, the NRPS PCR assay captured successfully several NRPS gene fragments in *E. coli* clones that matched corresponding NRPS genes on the GenBank. Those positive hits indicate the existence of NRPS biosynthetic genes within the soil metagenome. A similar PCR screening approach was used by other research groups to capture NRPS clones from soil metagenomes [28]. These findings are in agreement with another study that confirmed capturing NRPS genes in Cuban soil metagenome using NRPS PCR using different primer pairs [28].

We clarified that NRPS PCR assay recovered clones with similarity to sequences belonging to the *Delftia* genus within *Proteobacteria* phyla. Several related NRPS sequences were isolated from different sites suggesting a widespread environmental distribution of bacteria harboring NRPS genes coding for secondary metabolites. Similar results were recorded by a research study on the metagenomic library extracted from the soil in Cuba [28]. Several reports confirmed the biotechnological potential of the *Delftia* genus. For instance, a recent study has identified Delftibactin A (NRP) isolated from novel environmental *Delftia* spp. with potent antimicrobial activity against methicillin-resistant *Staphylococcus aureus*, vancomycin-resistant *Enterococcus*, *Acinetobacter baumannii*, and *Klebsiella pneumonia* [34]. Furthermore, the biotechnological perspective of *Delftia* sp. JD2 was previously confirmed using a genomic approach [35]. In the same context, functional NRPS genes were identified in *Delftia tsuruhatensis* MTQ3 for bacteriocins and siderophore production [36].

In the case of natural product science, phylogenetic relationships are highly informative in the design and function of the genes involved in secondary metabolite biosynthesis. Nonribosomal peptide synthetases provide a model in which individual domain phylogenies exhibit various predictive capabilities, determining features of substrate specificity correlated to the final metabolic product [37]. In this study, sequencing of selected NRPS clones showed that they belonged to different types of NRPS gene clusters; thus, they were separated into different phylogenetic trees. The difference in the nucleotide identity percentage and the presence of repeated DNA sequences in nearly all sequence clones can be due to mutations and confirm that they are diverse. It also may be evidence of the evolutionary process of NRPS genes [38]. Phylogenetic relationships showed that clones no. 15cd30 and 15cd34 were clustered together as a sister group to the genus *Delftia*, supported by high blast identity values, and the tree was in agreement with a previous phylogenetic analysis of the group [28]. Clone nos. 15cd30 and 15cd34 shared amino acid similarity with strains of *Delftia tsuruhatensis* and *Delftia acidovorans*, suggesting similar secondary metabolite production. However, they even have some mismatches at the terminal region indicating a distinct scope of bioactive molecule production. In the case of clone 15cd35 and clone 15cd37, NRPS gene sequences exhibited noticeable similarity values to AMP (A) domain sequences that belonged to members of genus *Delftia* spp., though they still showed several differences in amino acid sequences and low bootstrap value (54%) with the closest phylogenetic neighbors. Thus, the metagenomic NRPS genes are predicted to produce distinct nonribosomal peptides or suggesting many of the clones recovered came from undiscovered NRP pathways. These changes in amino acid sequences can be due to conservative mutation or radical substitution [41, 42]. In particular, the mutations are detected in the protein interior which may be a sign of structural constraints [43]. A similar study confirmed multiple alignments and the phylogenic tree of amino acid sequences of NRPS genes of *Streptomyces* sp. BDUSMP 02 reveals the potential to produce a new type of antibacterial compound belonging to the NRPS type [44]. Other studies supported using multiple alignments and phylogenetic tree approach in identifying conserved sequence regions and establishing evolutionary relationships [38]. Similarly, a study emphasized that sequence analysis of the 21 most active improved variants revealed that each contained between one and three changes to their primary amino acid sequence, suggesting that minimal sequence variation was able to effect dramatic improvements in NRPS domain function [45]. Fischbach et al. (2007) declared that NRPS is considered as enzymes that assemble the key skeletons of natural products, so any mutation of these genes is expected to give the clearest impact on the metabolite pattern and their functions. In agreement with our results, a substitutional mutation in NRPS genes can lead to ≈10-fold improvements in enzyme activity and can trigger the creation of new derivatives of NRP antibiotics [23].

In this study, bioinformatic tools such as NRPS predictor tool, NRPS predictive blast webserver, and PDBsum database were used to identify NRPS domains at both genome/proteome level and their substrate suggesting different structures with relevant biological activities. Similarly, other studies support the use of the NRPS predictor tool for reliable prediction of adenylation domain (NRPS) specificities [30, 46]. None of the predictive methods could infer any substrate specificity for 15cd35 and 15cd37 NRPS sequences, suggesting completely new types of specificity and PDBsum database showed their resemblance to acetyltransferase domains still with a low *z*-score. This may be due to mutations from corresponding sequences recorded in the multiple alignments. Different substrate specificity using bioinformatic tools refers to variation in the NRPS clones; thus, different nonribosomal peptides are biosynthesized. Similar results recorded that substrate specificity is important in determining the final bioactive product such as the A domain TycB_m3 activates l-tryptophan and phenylalanine for tyrocidine biosynthesis [31, 47]. Additionally, A domain of the barbamide biosynthetic gene cluster activates 100% specificity for leucine and valine and 80% for trichloroleucine [48].

Our colleague Dr. Chiara Borsetto, the University of Warwick, UK, had done a full characterization of adenylation domains of NRPS gene clusters from selected clones in the Cuban metagenomic library and integrate it into a BAC heterologous host system. However, her attempt was unsuccessful; this was due to the relatively small size of the metagenomic library (approximately 120 Mb) and shearing of the DNA due to inhibitory compounds during DNA extraction steps. This affects the efficiency of packaging and transduction into the *E. coli* host to obtain a larger library. However, the partial clusters recovered in our study are still of particular interest to attempting high-throughput transfer methods which will help in the characterization of the recovered clusters and future screening procedures [49]. Several hits of transposons with several repeats indicate the possibility of gene disruptions in the biosynthetic clusters which will lead to different metabolites and enrich the diversity and the produced metabolite. Transposons are called jumping genes that refer to large quantities of repetitive content in genomes by a process of horizontal gene transfer. They are known to affect transcriptional regulation in several different ways, and gene promoters are derived from transposons or have origins in transposon-induced duplication [50]. A recent study suggested that disruption of biosynthetic clusters can lead to different metabolic outcomes [32, 51]. Hit with several transposons indicates that the NRPS domains were more likely to have several adaptations that lead to a change in their diversity [32] and, consequently, will affect the compound produced.

Our results confirmed that the clone 15cd30 sequence was grouped with a similar protein sequence retrieved from compost metagenome, with cluster coding for gramicidin. This indicates that it may produce gramicidin like an antibiotic. Gramicidin is a pentadecapeptide antibiotic secreted by *Bacillus brevis* ATCC 8185 within the sporulation stage [52]. Gramicidin, also known as gramicidin D, is a mixture of ionophoricantibiotics including gramicidin A, B, and C, with a ratio of 80%, 5%, and 15%, respectively [53]. Gramicidins inhibit the growth of Gram-positive bacteria like *Bacillus subtilis* and *Staphylococcus aureus* [54]. Several mutations were represented in the branch length of clone 15cd30 sequence which indicates they are still different nucleotide substitutions over time, and consequently, we propose that it will produce a distinct metabolic product.

Finally, this study emphasizes the presence of biosynthetic NRPS genes in the soil metagenomic library. The molecular, phylogenetic, and bioinformatic analysis confirmed that mainly 15cd35 and 15cd37 are distinct clones harboring biosynthetic potential with undetected substrate specificity and thus can produce improved yield or even new antibiotics. Similar results confirmed that directed mutation in substrate specificity code within the A domain of NRPS genes can produce a high yield of certain antibiotics or even a novel antimicrobial compound [52–55]. This study supports the methodology of using PCR assay for screening soil metagenomes as a tool for drug discovery. Further genetic manipulation of NRPS clones will provide a positive impact on the pharmaceutical sector and consequently the health sector.

## Conclusion

In conclusion, the study highlights the presence of NRPS biosynthetic genes in the soil metagenomic library from Cuba. The results showed that the clones 15cd35 and 15cd37 have high biosynthetic potential and the potential to produce distinct antibiotics. The study supports the use of PCR assays for screening soil metagenomes as a tool for drug discovery, and genetic manipulation of these NRPS clones holds promise for the future of the pharmaceutical industry. Overall, this study highlights the diverse biosynthetic potential of Cuban soil and its potential to contribute to the development of new antibiotics and other bioactive compounds.

## Data Availability

Not applicable.

## Abbreviations

NRPS: nonribosomal peptide synthase
PKS: polyketide synthase
PDB: Protein Data Bank
HMM: hidden Markov model
AMP: adenosine monophosphate

## References

[1] World Health Organization (2020). Lack of new antibiotics threatens global efforts to contain drug-resistant infections.

[2] Kmietowicz Z (2017). Few novel antibiotics in the pipeline, WHO warns. BMJ: British Medical Journal (Online).

[3] Falagas ME, Lourida P, Poulikakos P, Rafailidis PI, Tansarli GS (2013). Antibiotic treatment of infections due to carbapenem-resistant Enterobacteriaceae: systematic evaluation of the available evidence. Antimicrobil agen chem AAC.

[4] Khanna M, Solanki R, Lal R (2011). Selective isolation of rare actinomycetes producing novel antimicrobial compounds. Int J Adv Biotechnol Res.

[5] Gillespie DE, Brady SF, Bettermann AD, Cianciotto NP, Liles MR, Rondon MR, Clardy J, Goodman RM, Handelsman J (2002). Isolation of antibiotics turbomycin A and B from a metagenomic library of soil microbial DNA. Appl Environ Microbiol.

[6] Amann RI, Ludwig W, Schleifer KH (1995). Phylogenetic identification and in situ detection of individual microbial cells without cultivation. Microbiol Mol Biol Rev.

[7] Bentley SD, Chater KF, Cerdeño-Tárraga AM, Challis GL, Thomson NR, James KD, Harris DE, Quail MA, Kieser H, Harper D, Bateman A (2002). Complete genome sequence of the model actinomycete Streptomyces coelicolor A3 (2). Nature.

[8] Hugenholtz P, Tyson GW (2008). Metagenomics. Nature.

[9] Escobar-Zepeda A, Vera-Ponce de Leon A, Sanchez-Flores A (2015). The road to metagenomics: from microbiology to DNA sequencing technologies and bioinformatics. Front Genet.

[10] Schloss PD, Handelsman J (2003). Biotechnological prospects from metagenomics. Curr Opin Biotechnol.

[11] Ginolhac A, Jarrin C, Gillet B, Robe P, Pujic P, Tuphile K, Bertrand H, Vogel TM, Perriere G, Simonet P (2004). Phylogenetic analysis of polyketide synthase I domains from soil metagenomic libraries allows selection of promising clones. Appl Environ Microbiol.

[12] Courtois S, Cappellano CM, Ball M, Francou FX, Normand P, Helynck G, Martinez A, Kolvek SJ, Hopke J, Osburne MS (2003). Recombinant environmental libraries provide access to microbial diversity for drug discovery from natural products. Appl Environ Microbiol.

[13] Carr R, Borenstein E (2014). Comparative analysis of functional metagenomic annotation and the mappability of short reads. PloS one.

[14] Marahiel MA, Stachelhaus T, Mootz HD (1997). Modular peptide synthetases involved in nonribosomal peptide synthesis. Chem rev.

[15] Wang H, Fewer DP, Holm L, Rouhiainen L, Sivonen K (2014). Atlas of nonribosomal peptide and polyketide biosynthetic pathways reveals common occurrence of nonmodular enzymes. Proceed Nat Acad Sci.

[16] Dejong CA, Chen GM, Li H, Johnston CW, Edwards MR, Rees PN, Skinnider MA, Webster AL, Magarvey NA (2016). Polyketide and nonribosomal peptide retro-biosynthesis and global gene cluster matching. Nat chem biol.

[17] Finking R, Marahiel MA (2004). Biosynthesis of nonribosomal peptides. Annu Rev Microbiol.

[18] Sieber SA, Marahiel MA (2005). Molecular mechanisms underlying nonribosomal peptide synthesis: approaches to new antibiotics. Chem rev.

[19] Cane DE, Walsh CT, Khosla C (1998). Harnessing the biosynthetic code: combinations, permutations, and mutations. Science.

[20] Cane DE, Walsh CT (1999). The parallel and convergent universes of polyketide synthases and nonribosomal peptide synthetases. Chem & biol.

[21] Amin DH, Tolba S, Abolmaaty A, Abdallah NA, Wellington EM (2017). Phylogenetic and antimicrobial characteristics of a novel Streptomyces sp. Ru87 isolated from Egyptian soil. Int J Curr Microbiol App. Sci.

[22] Amin DH, Abolmaaty A, Tolba S, Abdallah NA, Wellington EM (2017). Phylogenic characteristics of a unique antagonistic Micromonospora Sp. Rc5 to S. aureus isolated from Sinai Desert of Egypt, Cur. Res Microbiol and Biotech.

[23] Fischbach MA, Lai JR, Roche ED, Walsh CT, Liu DR (2007). Directed evolution can rapidly improve the activity of chimeric assembly-line enzymes. Proceed Nat Acad Sci.

[24] Komaki H, Harayama S (2006). Sequence diversity of type-II polyketide synthase genes in Streptomyces. Actinomycetologica.

[25] Amin DH, Borsetto C, Tolba S, Abolmaaty A, Abdallah NA, Wellington EM (2017). Phylogenic analysis of NRPS and PKS genes associated with antagonistic Micromonospora Rc5 and Streptomyces Ru87 isolates. J Adv Biology & Biotechnology.

[26] Amin DH, Abolmaaty A, Tolba S, Abdallah NA, Wellington EM (2017). Phylogenic characteristics of a unique antagonistic Micromonospora Sp. Rc5 to S. aureus isolated from Sinai Desert of Egypt. Cur Res Microbiol and Biotech.

[27] Kallifidas D, Kang HS, Brady SF (2012). Tetarimycin A, an MRSA-active antibiotic identified through induced expression of environmental DNA gene clusters. J Ameri Chem Soc.

[28] Amos GC, Borsetto C, Laskaris P, Krsek M, Berry AE, Newsham KK, Calvo-Bado L, Pearce DA, Vallin C, Wellington EM (2015). Designing and implementing an assay for the detection of rare and divergent NRPS and PKS clones in European, Antarctic and Cuban soils. PloS one.

[29] Ayuso-Sacido A, Genilloud O (2005). New PCR primers for the screening of NRPS and PKS-I systems in actinomycetes: detection and distribution of these biosynthetic gene sequences in major taxonomic groups. Microbiol ecol.

[30] Miller G, Lipman M (1973). Release of infectious Epstein-Barr virus by transformed marmoset leukocytes. Proceed Nat Acad Sci.

[31] Mootz HD, Marahiel MA (1997). The tyrocidine biosynthesis operon of Bacillus brevis: complete nucleotide sequence and biochemical characterization of functional internal adenylation domains. J bacteriol.

[32] Mootz HD, Kessler N, Linne U, Eppelmann K, Schwarzer D, Marahiel MA (2002). Decreasing the ring size of a cyclic nonribosomal peptide antibiotic by in-frame module deletion in the biosynthetic genes. J Amer Chem Soc.

[33] Hall TA (1999). BioEdit: a user-friendly biological sequence alignment editor and analysis program for Windows 95/98/NT.

[34] Tejman-Yarden N, Robinson A, Davidov Y, Shulman A, Varvak A, Reyes F, Rahav G, Nissan I (2019). Delftibactin-A, a non-ribosomal peptide with broad antimicrobial activity. Front in Microbiol.

[35] Morel MA, Iriarte A, Jara E, Musto H, Castro-Sowinski S (2016). Revealing the biotechnological potential of Delftia sp. JD2 by a genomic approach. AIMS Bioeng.

[36] Guo H, Yang Y, Liu K, Xu W, Gao J, Duan H, Du B, Ding Y, Wang C (2016). Comparative genomic analysis of Delftia tsuruhatensis MTQ3 and the identification of functional NRPS genes for siderophore production. BioMed Res Intern.

[37] Ziemert N, Jensen PR (2012). Phylogenetic approaches to natural product structure prediction. Meth in enzym.

[38] Wang L, Jiang T (1994). On the complexity of multiple sequence alignment. Journal of comput bio..

[39] Thompson JD, Higgins DG, Gibson TJ (1994). CLUSTAL W: improving the sensitivity of progressive multiple sequence alignment through sequence weighting, position-specific gap penalties and weight matrix choice. Nucl acids res.

[40] Tamura K, Stecher G, Peterson D, Filipski A, Kumar S (2013). MEGA6: molecular evolutionary genetics analysis version 6.0. Mol biol evol.

[41] Zhang J (2000). Rates of conservative and radical nonsynonymous nucleotide substitutions in mammalian nuclear genes. J mol evol.

[42] Dagan TY, Graur D (2002). Ratios of radical to conservative amino acid replacement are affected by mutational and compositional factors and may not be indicative of positive Darwinian selection. Mol biol evol.

[43] Sivalingam P, Muthuselvam M, Pote J, Prabakar K (2019). Phylogenetic insight of nonribosomal peptide synthetases (NRPS) adenylate domain in antibacterial potential Streptomyces BDUSMP 02 isolated from Pitchavaram Mangrove. Bioinformation.

[44] Owen JG, Calcott MJ, Robins KJ, Ackerley DF (2016). Generating functional recombinant NRPS enzymes in the laboratory setting via peptidyl carrier protein engineering. Cell chem biol.

[45] Rausch C, Weber T, Kohlbacher O, Wohlleben W, Huson DH (2005). Specificity prediction of adenylation domains in nonribosomal peptide synthetases (NRPS) using transductive support vector machines (TSVMs). Nucl acids res.

[46] Agüero-Chapin G, Pérez-Machado G, Sánchez-Rodríguez A, Santos MM, Antunes A (2016). Alignment-free methods for the detection and specificity prediction of adenylation domains. Nonribosomal Peptide and Polyketide Biosynthesis.

[47] Chang Z, Flatt P, Gerwick WH, Nguyen VA, Willis CL, Sherman DH (2002). The barbamide biosynthetic gene cluster: a novel marine cyanobacterial system of mixed polyketide synthase (PKS)-non-ribosomal peptide synthetase (NRPS) origin involving an unusual trichloroleucyl starter unit. Gene.

[48] Stachelhaus T, Mootz HD, Marahiel MA (1999). The specificity-conferring code of adenylation domains in nonribosomal peptide synthetases. Chem & biol.

[49] Borsetto C (2017). Study and exploitation of diverse soil environments for novel natural product discovery using metagenomic approaches.

[50] Edlefsen PT, Liu JS (2010). Transposon identification using profile HMMs. BMC Genomics. b.

[51] Phelan VV, Moree WJ, Aguilar J, Cornett DS, Koumoutsi A, Noble SM, Pogliano K, Guerrero CA, Dorrestein PC (2014). Impact of a transposon insertion in phzF2 on the specialized metabolite production and interkingdom interactions of Pseudomonas aeruginosa. J Bacteriol May.

[52] Hotchkiss RD, Dubos RJ (1940). Fractionation of the bactericidal agent from cultures of a soil Bacillus. J Biolog Chem.

[53] Stankovic CJ (1990). *1.* A two-directional chain synthesis approach to 6-deoxyerythronolide B. 2. Design, synthesis, and analysis of new ion channels based on the gramicidin A motif.

[54] Kessler N, Schuhmann H, Morneweg S, Linne U, Marahiel MA (2004). The linear pentadecapeptide gramicidin is assembled by four multimodular nonribosomal peptide synthetases that comprise 16 modules with 56 catalytic domains. J Biolog Chem.

[55] Symmank H, Franke P, Saenger W, Bernhard F (2002). Modification of biologically active peptides: production of a novel lipohexapeptide after engineering of Bacillus subtilis surfactin synthetase. Protein engr.

